# No Retrenchment From Employee Empowerment: Employer Wellness Imperatives and Opportunities Emerging From the COVID-19 Pandemic

**DOI:** 10.3389/fpubh.2022.918784

**Published:** 2022-07-19

**Authors:** George A. Gellert, Scott Montgomery, Oliver Bridge, Tess E. Gellert

**Affiliations:** ^1^Evidence-Based Solutions, San Antonio, TX, United States; ^2^Wellteq, Singapore, Singapore; ^3^Wellteq, Melbourne, VIC, Australia; ^4^Wellteq, Vancouver, BC, Canada

**Keywords:** employee wellness, occupational health, COVID-19 pandemic, employee empowerment, post-COVID-19 employer wellness challenges and imperatives

## Abstract

The impact of two years of the COVID-19 pandemic on the relationship between employers and employees are explored, including changing employee sensibilities with respect to future employment, work-life balance, remote and flexible work, and the great resignation. Lasting work changes induced by the pandemic expand employee empowerment and demand for greater work flexibility. Flexibility no longer provides employers a unique selling point and hiring/retention competitiveness – it has become an expected standard. Evolving workplace expectations are tied to realizations of the value of work within the broader context of employees' lives, changing business culture across many industries. Demand for increased work/employment individualization and personalization overlaps unprecedented personalization of and power of mobile technologies. Human-centered employee management in the post-COVID-19 era will become imperative, with many opportunities for employers to enable greater impact in employee wellness and health promotion driven by deploying compelling virtual-remote engagement and behavioral change technologies.

## Introduction: Post-Pandemic Sensibilities in Enterprise Employee Health

Few leaders of employee health efforts are sorry to bid farewell to 2021. The continued impact of the COVID-19 pandemic has been felt far and wide, and as individuals have re-evaluated their work life and career priorities, many in employee health are observing what has been described as “the great resignation,” in which individuals are leaving jobs for new work and lifestyle opportunities brought by the pandemic (1–3). At the same time, a silver lining resulting from the pandemic has been an enormous acceleration in the acceptance and adoption of remote work among employees across many industries, as well the engagement of employee telehealth and remote wellness, support and monitoring technologies (4). This dramatic shift is forcing businesses already struggling to adapt and evolve operations in the pandemic to also keep pace and adopt these new technologies in order to meet employee expectations and needs (2, 3).

After enduring a second year of the global pandemic, employees in many if not most industries are seeking respite from the uncertainty it has created in their personal as well as their work lives. While the coming years will no doubt bring their own set of employee wellness challenges, there will also be significant opportunities. Employers agile enough to embrace these pandemic-induced changes in technology use, organizational culture, employer–employee dynamics, and efforts to maximize employee health and wellness will adapt, evolve and thrive. Enterprises which cannot or will not accept and adapt to these changes in the emerging post-pandemic norm may not be as fortunate. It is critical for employers to understand what these changes mean for the future of employee health and wellness, and thus to their core business. So, what can employers and the employer wellness industry expect in the coming year?

## Pandemic Induced Work Flexibility and Engaging Employee-Driven Organizational Change

Businesses across the globe have been brutalized by the last two pandemic years (5). For many, the goal has been to mitigate revenue loss and survive financially. Ones that could progress toward remote work by engaging, enabling and psychologically supporting their employees from afar, have done well. For others, it has been an extremely difficult and troublesome period (5). A demonstration of how businesses have transformed is seen in how many have gone from a history of largely face-to-face, nine-to-five, clock-in-clock-out work models to almost literally overnight embracing work from home, flexible hours, and self-reporting of time worked (6). Soon thereafter came the challenge of the great resignation of employees, along with the adaptation of employers who increasingly allowed people to work in roles, styles and at organizational levels that were not previously offering such flexible career paths. This can potentially set back or advance individual companies and conceivably entire industries.

Industries can maximize opportunities in this time of great flux. Clearly for many employees, COVID-19 has caused individuals to reflect about what is of greatest importance in their lives. Employers will need to be deliberate and systematic about engaging and not ignoring these employee realizations. It would be unwise to just return to what was. At this historical juncture, during this pandemic and in the post-pandemic era, companies may need to embrace what is important to their employees, and what is essential to their success in this new business environment, or they may fall behind. What can businesses do to thrive in the post-pandemic world?

The successful shift to remote working during the pandemic likely means that for many employees, flexible work arrangements and hours are here to stay (7). However, while some employees thrive in this new environment, others do not. Other employees work in industries and positions, such as in the large services economy, retail sales and manufacturing, where remote work is not an option for many. For employees in these sectors who would value remote work, if feasible, employers would do well to explore hybrid opportunities that mix on site work with remote work. Indeed, this may provide opportunities to grow the skills and contribution of employees through diversification of their duties and contribution to the organization. Employers will need to work with employees to create work arrangements considering individual factors. Sustained adoption of a hybrid work model will become the norm in many industries, rather than the exception (8). Remote communication has enabled business continuity in the greatest global public health crisis in over a century. Many employers have been creative in enabling and empowering people to find new ways of delivering value remotely. However, the approach taken by most has been primarily reactive and stopgap rather than proactive and creative. To succeed over the long term, this must change.

The pandemic has changed not only the way employees work, but it has fundamentally altered how employees feel about work. The pandemic has caused people (and employees) to re-examine their lives and basic values (9). After enduring a global pandemic, many have come to recognize that life can no longer be taken for granted. As a result, employees increasingly hunger for a more balanced lifestyle – and this is contributing to the great resignation (1–3). The term “resignation” has dual meaning, because employees are not only resigning from a particular employed position, but also undergoing an emotional and psychological process of resigning from their existing lifestyles to seek new and different ways of living and being employed, along with a new set of values and sensibilities. In this sense, the great resignation is, at an individual employee level, a major personal transformation occurring among millions of people.

In turn the great resignation is having a transformative effect on the workplace. Employers are finding many employees wanting more from their workplaces, and no longer just accepting outdated models of business culture. This is upending a long period of power asymmetry between employers and their employees, where employers directed employees on all key workflow decisions and processes, with employees simply complying. In an unprecedented shift, employees are demanding that they be treated as individuals with very diverse and different requirements. As a result, the coming era will be defined by a need for greater employer flexibility and adaptability (8, 9). It is interesting and likely no coincidence that the great resignation and demand for increased work/employment individualization and personalization is occurring at the same time as an unprecedented personalization of mobile technologies, and within health care and medical fields (10).

Flexibility will no longer provide employers with a unique selling point and hiring or retention competitiveness – rather it has become an expectation and an evolving standard. In hybrid employment arrangements, in-person/in-office work will persist, but employers will need to be more deliberate about when it occurs, rather than being an everyday standard. Moving forward, work arrangements will not be determined solely by employer preferences. Compromise and mutual accommodation in work arrangements with employees will become increasingly common, and the best process and outcomes will not be defined only by the business but also employees. This may be relatively difficult for old-school and highly traditional managers to adopt and adapt to, but can be more feasible for modern-day people leaders. Promoting a culture of open and frank conversation, genuine trust and ongoing dialogue with employees about what they need is becoming a competitive hiring, retention, productivity and employee wellness standard.

## Human-Centered Employee Management in the Post-COVID-19 Era

There is undeniably a human dimension of business that has historically been somewhat subordinated, but that companies now need to focus upon as an essential business imperative. The shift in employee expectations is leading to an unprecedented demand for a human-centered approach to structural workplace design, workflows and brand purpose (11). The digital-first generation of employees tend to be more purpose- and goal-driven than their predecessors. If organizations can genuinely align how their people are enabled to work and give them a purpose, and a sense of professional meaning, then employees are more empowered, aligned with business objectives, and satisfied. In the pandemic and post-pandemic era, employers need more than ever to shift to a people-centric focus within business teams in the context of what is being produced and why. If employers can foster creative thinking and problem solving, then combine this with purpose-driven foundations and flexibility – this will recognize and leverage the changes in employee life orientation induced by the pandemic and enable employers to positively leverage their employees' passions and capabilities.

To further enhance and enrich the employee experience, leaders must look beyond job roles, titles and performance metrics and try to envision how they can optimally support their people - not just in day-to-day work processes but in career planning and progression, ongoing professional training, succession planning, more collaborative working styles, development of new initiatives of importance to the organization, and welcoming and fostering creativity and innovation. Equally important will be that employers take an active role in helping their employees to achieve an effective and personally satisfying work-life balance. In the post-pandemic great resignation, business planning and strategic decisions need to be developed and assessed with a people-first lens on (11).

Most every organization has in the past embraced the rhetoric of “our people are our most important asset,” but in the post-pandemic era, employers will have to demonstrably prove this and will have to “walk the talk” with their employees as never before. Employee-centered practices, policies and benefits will need to be impactful and measurable, and clearly a recognizable value and orientation within the organization's culture. This will be critically important for companies who not only want to retain and recruit talent, but for those who seek to attract and retain the very best employees.

## Lemons Into Lemonade: Seizing the Post-Pandemic Employee Wellness and Health Promotion Opportunity

Given the persistent global health crisis, an essential focus for businesses in the coming years will be to prioritize the wellness and health outcomes of their employees. Health and wellbeing will play an outsized role in generating and maintaining a successful and productive workplace culture as a lasting impact of the pandemic. As more and more employees opt for flexible work conditions, organizational leaders will need to re-evaluate how employee health and wellness can be most effectively managed and supported.

Employers will need to lead by “putting the H” back into organizational WHS (Workplace Health & Safety) and occupational Health, Safety and Environment (HSE). Historically in Australia, safety has been embraced by employers, but what about the health and wellness part of this strategy and commonly used acronym? We have been somewhat absent in looking after our employees' health, especially in Australia, where the corporate wellness market often receives a fraction of the spend in the occupational safety market (12, 13). In recent years, Australian investment in health and safety programs has been about $2.1 billion, while corporate wellness program spend has been $287 million (12, 13). We need to evolve and become much better at understanding and meaningfully engaging our employees' wellbeing, and organizations will to need develop and implement an individualized approach to employees in order to best support them, both as employees and more generally and beyond the workplace as individuals.

The challenge for many enterprise leaders will be building the trust with employees that enable honest conversations about health and wellbeing, and the engagement that will facilitate employees getting into the care pathways they need. Many employees are reluctant to seek out help. Australia has had employee assistance programs (EAPs) for over 30 years, but the average utilization still only sits somewhere between 5 and 7% in most organizations (14). This is the case despite the fact that any time, at least 20% of the employee population should be having conversations with a mental health or psychosocial support professional around a variety of issues and concerns (14). This leads us to question what is happening to the remaining 13%−15% of the employee population that currently is not accessing an EAP but should be/needs to? And what percentage of that underserved group ends up experiencing preventable work absences, engaged in avoidable and costly health care utilization, or preventable loss of work productivity and quality?

This is where our focus should be, and unfortunately that percentage of health and wellness underserved employees has been growing for years, and is now accelerated by the pandemic and all of its diverse and pernicious fallout. This is where our great opportunity sits as employers. We need to find more effective and more engaging ways to humanize employee health management and more compellingly provide psychosocial wellness and support opportunities. Here the promise and reality of existing technology can help support employees and improve their health and wellness status and outcomes with unprecedented cost-effectiveness. Having an overall framework and specific psychosocial support delivery vehicles in place are vital for any enterprise seeking to take the first steps in transforming their employees' health and wellness journey. Provision of engaging psychosocial support is a critical and integral element in compelling employees to make behavioral changes that reduce health and wellness risk, and where early intervention and prevention opportunities can be first realized (15).

This convergence and transformation of employee health awareness and health care in the workplace is only just beginning. Employers and employees share a common interest in disease prevention, physical and mental health risk reduction and care utilization and cost avoidance for both acute, short-term and chronic long-term illness, and for both physical ailments and the mental health problems we have seen increase so dramatically over the course of the pandemic. These mental health challenges, including acute stress, anxiety and depression, will likely persist well beyond when COVID-19 has diminished from a pandemic to an endemic and routinely managed component of our healthcare delivery system, just like the HIV/AIDS pandemic before it (16).

Remote engagement and personalized technology that helps employees better understand and more effectively engage their own health promotion and self-care is the future of employee health and wellness, and will enable greater prevention of avoidable health issues and outcomes that negatively impact employees and employers equally (17–20). The cost-effective leveraging of employee engagement and behavioral change technology not only promotes healthy living/risk reduction and wellness, but can increase employee workplace engagement and productivity, as well as job satisfaction (18).

## A Brave New World of Employee Health and Wellness Promotion Driven by Compelling Virtual-Remote Engagement and Behavioral Change Technology

After so much upheaval across so many different industries, it may be difficult to imagine that there are likely more to come before this viral plague becomes just one of many reportable and manageable infectious diseases. But the new year brings new opportunities, many of which are increasingly delivered by rapidly evolving technologies that enable a high degree of employee personalisation and which effectively drive employee behavior change, risk reduction and deep employee engagement (19, 20). Organizations can clearly and demonstrably benefit from such innovative personalized technology for their employees and enable them to focus on what really matters in terms of achieving a truly positive and sustainable impact on their employees.

However, given the business and financial pressures resulting from the pandemic, now is not the time to deviate too far from one's strategic business vision and pathways. But employers can readily integrate compelling and high impact technology into the fabric of their organization and the health and wellness journey of their employees (19, 20). Current technologies and platforms are remarkable both with respect to their power to change employee behavior and their affordability. Many are demonstrably valuable, but most businesses are only now catching up. Employers need wellness companies that truly understand how to practice collaborative partnership in order to effectively harness these technologies. Software, algorithmic artificial intelligence, and machine learning capabilities will catapult us into the future, but all still require a human understanding and application within the workplace.

The number of employee health and wellness improvement opportunities that have been made affordable by virtual and remote technology has increased dramatically throughout the pandemic. The imperative now is not to squander these opportunities or revert to a pre-pandemic mode and sense of urgency to improve employee health and wellness. We are potentially entering a golden era in the advancement of employee health and wellness promotion and risk reduction, and this is not just because of the power of virtual health engagement, remote technology and telemedicine. While the degree of sophistication in technology continues to advance formidably, it is also being deployed in ways we had never imagined even a decade ago to increase employee health promotion impact and reduce avoidable care utilization and costs.

For employers, a concomitant opportunity exists to engage changes in organizational design to drive deeper employee work purpose and satisfaction. Efforts to leverage and apply the latest innovations in virtual and remote employee engagement will be facilitated if employers, understanding their local market and employee cultures, consider the influence of longstanding local practices, culture and regulations. Proactively considered and attended to, these can facilitate the ongoing virtual-remote transformation of the workplace; ignored, they will likely impede it.

As occurs frequently with the introduction of new disruptive technologies, while many workers will be able to take advantage of the opportunities, no single technology is a panacea, and historically technologically disenfranchised segments of any community, including within any company, who have less technology literacy or personal access to current state technology, may not benefit to the extent of others in this evolving environment. Employers would do well to consider these factors, and for both corporate financial stewardship and to maximize employee health and wellness, should endeavor to ensure equity for these employee segments, where the impact of such new technologies may be much needed, substantial and valuable.

There are already over 25,000 healthcare applications for the iPhone, and it and other smartphones and tablets have become powerful diagnostic devices, as well as vehicles for achieving substantial and sustainable improvements in health outcomes and reducing employees' health and wellness risks. When combined with wearables embedded within an integrated employee engagement platform, the power to impact and achieve improvements in employee health and wellness increase exponentially. These applications and technologies are our best defense to slow and then reduce ever increasing employee risk, whether individuals are suffering from anxiety and high stress, depression, chronic insomnia and obstructive sleep apnea, or diabetes, hypertension, cardiovascular disease and stroke. With such diverse specialization employers will need a concierge function to help navigate an increasingly complex and potentially overwhelming ecosystem.

As virtual employee engagement and behavioral change technology continues to advance, it will reduce health risk and disease incidence, saving lives in excess of those saved by existing health care delivery that is focused less on prevention and more on mitigating impact once disease exists. In reducing avoidable health risks and preventing health problems, including employee burnout and broader mental health, employers will have a substantial opportunity to support their employees' wellbeing and enhance their work-life balance better than has been hitherto possible. The future of employee health and wellness promotion has arrived, partly accelerated by a terrible plague, and that future is virtual, technology-mediated and will impact and deliver value across the employee health lifecycle at home, work and play.

## Author Contributions

Conceptualization and writing: GG, SM, OB, and TG. Literature research: TG and GG. All authors contributed to the article and approved the submitted version.

## Conflict of Interest

GG is a medical advisor to Wellteq. SM, OB, and TG were employed by Wellteq.

## Publisher's Note

All claims expressed in this article are solely those of the authors and do not necessarily represent those of their affiliated organizations, or those of the publisher, the editors and the reviewers. Any product that may be evaluated in this article, or claim that may be made by its manufacturer, is not guaranteed or endorsed by the publisher.
